# Identification and verification of novel immune-related ferroptosis signature with excellent prognostic predictive and clinical guidance value in hepatocellular carcinoma

**DOI:** 10.3389/fgene.2023.1112744

**Published:** 2023-08-21

**Authors:** Wenxiu Jiang, Lili Wang, Yajuan Zhang, Hongliang Li

**Affiliations:** ^1^Department of Infectious Diseases, The People’s Hospital of Danyang, Affiliated Danyang Hospital of Nantong University, Danyang, China; ^2^Department of Clinical Research, The Second Hospital of Nanjing, Nanjing Hospital Affiliated to Nanjing University of Traditional Chinese Medicine, Nanjing, China; ^3^ General Medicine, Pingjiang Xincheng Community Health Service Center, Suzhou, China

**Keywords:** immune-related ferroptosis, signature, prognostic predictive value, clinical value, hepatocellular carcinoma

## Abstract

**Background:** Immunity and ferroptosis often play a synergistic role in the progression and treatment of hepatocellular carcinoma (HCC). However, few studies have focused on identifying immune-related ferroptosis gene biomarkers.

**Methods:** We performed weighted gene co-expression network analysis (WGCNA) and random forest to identify prognostic differentially expressed immune-related genes (PR-DE-IRGs) highly related to HCC and characteristic prognostic differentially expressed ferroptosis-related genes (PR-DE-FRGs) respectively to run co-expression analysis for prognostic differentially expressed immune-related ferroptosis characteristic genes (PR-DE-IRFeCGs). Lasso regression finally identified 3 PR-DE-IRFeCGs for us to construct a prognostic predictive model. Differential expression and prognostic analysis based on shared data from multiple sources and experimental means were performed to further verify the 3 modeled genes’ biological value in HCC. We ran various performance testing methods to test the model’s performance and compare it with other similar signatures. Finally, we integrated composite factors to construct a comprehensive quantitative nomogram for accurate prognostic prediction and evaluated its performance.

**Results:** 17 PR-DE-IRFeCGs were identified based on co-expression analysis between the screened 17 PR-DE-FRGs and 34 PR-DE-IRGs. Multi-source sequencing data, QRT-PCR, immunohistochemical staining and testing methods fully confirmed the upregulation and significant prognostic influence of the three PR-DE-IRFeCGs in HCC. The model performed well in the performance tests of multiple methods based on the 5 cohorts. Furthermore, our model outperformed other related models in various performance tests. The immunotherapy and chemotherapy guiding value of our signature and the comprehensive nomogram’s excellent performance have also stood the test.

**Conclusion:** We identified a novel PR-DE-IRFeCGs signature with excellent prognostic prediction and clinical guidance value in HCC.

## Introduction

Liver cancer has become the sixth most common cancer and third leading cause of cancer-related deaths worldwide (Sung et al., 2021). As the most common subtype in primary liver cancer, the attack rate of hepatocellular carcinoma (HCC) has tripled in the past 3 decades (Altekruse et al., 2009). Although studies over the past half-century have tried to reveal the epidemiology, pathogenic factors, and genetic characteristics of HCC, which have contributed to advancing the improvement of its early prevention, diagnosis, and therapy strategies, most patients remain in the middle and late stages of the disease (Tomaz et al., 2015; Bertuccio et al., 2017; Llovet et al., 2018; Petrick et al., 2020). Therefore, the prognosis of HCC treated with surgery, chemotherapy and radiotherapy is not ideal (Jemal et al., 2017). Statistically, 70% and more than 90% of HCC recurrences occur within 2 and 5 years after surgery, respectively, which are associated with poor response to treatment and lower survival rates (Zheng et al., 2017). Therefore, it is urgent to identify novel genetic signature closely related to HCC’s occurrence and progression with high prognostic prediction accuracy and therapeutic guidance value.

The immune system, including immune cells, immune factors and immune microenvironment, has been proved to be an important factor in tumorigenesis (Sima et al., 2019). Tumor-associated immunity, whose effects include disruption of genome stability, obvious genetic modification, promotion of tumor cell proliferation, resistance to tumor apoptosis, stimulation of angiogenesis, and shaping of tumor microgrowth environment, exists in all stages of tumorigenesis (Gonzalez et al., 2018; Yang et al., 2021a). As an emerging therapeutic approach in the field of cancer therapy in recent years, immune checkpoint inhibitor (ICI) has demonstrated strong antitumor activity in many cancers (Bronte et al., 2010; Mellman et al., 2011; Khalil et al., 2016; Liu et al., 2018). In particular, ICI such as programmed death 1 (PD-1) and programmed death ligand 1 (PDL-1) have shown good therapeutic response in the clinical first-line treatment of HCC (Harding et al., 2016). The CheckMate 040 trial showed that Nivolumab had a control rate of about 60% in patients with HCC that had progressed after standard sorafenib therapy (Harding et al., 2016). The KEYNOTE 240 trial also confirmed that more than two-thirds of HCC patients receiving sorafenib responded to Pembrolizumab (Zhu et al., 2018; Hong et al., 2020). However, the proportion of HCC patients who benefit from ICI treatment is still very limited as many factors, such as immune system and tumor immune microenvironment (TIME), can affect the ICIs’ efficacy (Nishino et al., 2017).

Ferroptosis, a new form of regulated cell death, differs from programmed cell death and is driven by iron-dependent peroxidation of lipids (Dixon et al., 2012; Zhuo et al., 2020). At present, the important role of ferroptosis in the inhibition of many cancers, including breast cancer (Kaplan and Ng, 2017), pancreatic cancer (Tang et al., 2020), ovarian cancer (Ye et al., 2021) and HCC (Shan et al., 2020; Deng et al., 2021), has been confirmed by many studies. In HCC, targeted ferroptosis related genes can further regulate the cancer cells’ growth by changing the cancer cells’ sensitivity to ferroptosis (Louandre et al., 2015; Jennis et al., 2016). For example, TP53 can make hepatoma cells sensitive to ferroptosis and inhibit their growth through SLC7A11 (Sun et al., 2016). UBA1 has also been reported to promote HCC’s development by up-regulating Nrf2 signal pathway and down-regulating Fe^2+^ levels (Shan et al., 2020). More and more studies have found that activation of ferroptosis in tumors has gradually become a new strategy for cancer treatment, especially for these resistant to conventional therapy (Hassannia et al., 2019; Mou et al., 2019; Luo et al., 2021). The activation of ferroptosis has also been shown to contribute to the efficacy of cancer treatment, such as ICI and radiotherapy (Friedmann Angeli et al., 2019; Wang et al., 2019; Lei et al., 2020; Song et al., 2021). It is worth mentioning that the process of ferroptosis in tumors has been observed to be associated with the immune microenvironment, implying that there is often a synergistic interaction between ferroptosis and immunity in tumor’s progression (Stockwell et al., 2020; Jiang et al., 2021). These results all suggest that novel immune-related ferroptosis gene signature have great potential in predicting prognosis and guiding clinical treatment of HCC.

With the continuous development of the computer field, numerous novel algorithms focus on identifying genetic markers that are closely related to diseases. As one of them, weighted gene co-expression network analysis (WGCNA) is often used to describe the correlation between genes in various cancer microarray tissues, to find modules that are highly related to the traits of external tissues, and to screen candidate biomarkers or therapeutic targets (Langfelder and Horvath, 2008; Giulietti et al., 2018; Nomiri et al., 2022). As one of the best traditional machine learning methods based on integrated learning principle (Sessa et al., 2020; Douville et al., 2021), random forest model shows high prediction accuracy in a large number of previous modeling, and provides more variable importance estimation than classifier (Tran et al., 2019). This study aims to use these advanced machine learning algorithms to screen prognostic differentially expressed immune-related ferroptosis characteristic genes (PR-DE-IRFeCGs) highly related to HCC, and to identify genes signature that can accurately predict the HCC cases’ prognosis and treatment response.

## Materials and methods

### Data acquisition sources and corresponding processing

**FIGURE 1:**
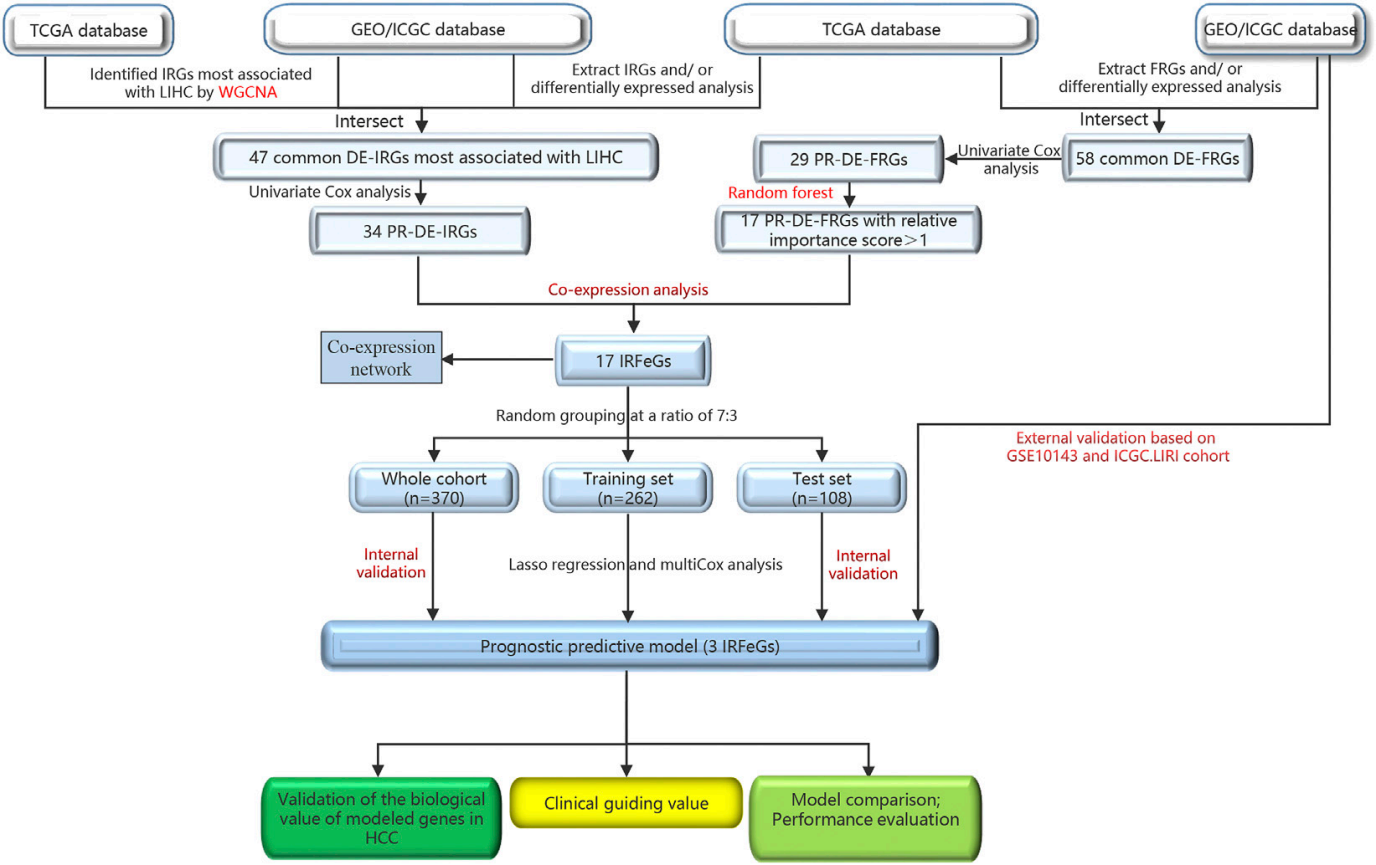
The entire flow of this study.

Figure 1 outlined the entire flow of this study. The Cancer Genome Atlas (TCGA, cancergenome.nih.gov/) database, International Cancer Genome Consortium (ICGC, dcc.icgc.org/projects/ORCA-IN) database and Gene Expression Omnibus (GEO, ncbi.nlm.nih.gov/geo) database provided the HCC-related RNA sequencing and clinical data. TCGA covers a HCC cohort containing 374 HCC and 50 adjacent normal tissues. We obtained GSE36376 cohort (193 HCC and 240 adjacent normal tissues), GSE14520 cohort (247 HCC and 241 adjacent normal tissues), GSE25097 cohort (268 HCC and 243 adjacent normal tissues) and GSE10143 cohort (80 HCC tissues) from GEO. LIRI cohort (273 HCC and 203 adjacent normal tissues), the last external cohort, was obtained from the ICGC (Liu et al., 2019; Yang et al., 2021b; Jin et al., 2022; Li et al., 2022). ImmPort (immport.org/home) and InnateDB (innatedb.ca/) databases provided 2,660 immune-related genes (IRGs), while FerrDb (zhounan.org/ferrdb) database shared 259 ferroptosis-related genes (FRGs) for us. Next, we obtained the sequencing value for the following genes: 1.247, 237, 193, 218, 140, and 242 FRGs from the TCGA cohort, GSE36376, GSE14520, GSE25097, GSE10143, and ICGC. LIRI cohorts, respectively; 2.2,366, 1,984, 1,528, 1,806, 1,167, and 1,983 IRGs from the TCGA cohort, GSE36376, GSE14520, GSE25097, GSE10143, and ICGC. LIRI cohorts, respectively.

### Identification of PR-DE-IRFeCGs

The differentially expressed immune-related genes (DE-IRGs) from the TCGA HCC cohort was performed under the filtering condition of | log2 fold change | (| log2FC |) > 0.585 and false discovery rate (FDR) < 0.05. After setting FDR <0.05 as the new filtering condition, we identified differentially expressed ferroptosis-related genes (DE-FRGs) and DE-IRGs from GSE36376, GSE14520, GSE25097, and ICGC cohorts as well as DE-FRGs from TCGA cohort. Next we extracted the common DE-IRGs and DE-FRGs from all the cohorts.

We ran WGCNA based on the IRGs’ sequencing value from TCGA, GSE36376, GSE14520, GSE25097, and ICGC cohorts separately for identifying the corresponding DE-IRGs most relevant to HCC. The specific processes were as follows: 1) After clustering the tissues from each cohort and excluding free tissues, the “pickSoftThreshold” function was used to select the best soft power β to build the best scale-free network (Fan et al., 2022a). 2) The adjacency matrix was created according to the formula:

$$aij=\left|Sij\right|\beta$$

( aij : adjacency matrix between gene i and gene j, Sij : similarity matrix which is done by Pearson correlation of all gene pairs, *β*: softpower value) (Zhu et al., 2021; Fan et al., 2022a). 3) We transformed the adjacency matrix into a topological overlap matrix and the corresponding dissimilarity (1-TOM) (Zhu et al., 2021; Fan et al., 2022a). 4) We aggregated highly correlated genes at 1-TOM distances to construct corresponding modules to match corresponding dynamic branches, and merge similar modules (Zhu et al., 2021; Fan et al., 2022a). Then, the common IRGs were extracted from the modules most relevant to HCC from each cohort (Zhu et al., 2021; Fan et al., 2022a). Similarly, we extracted common DE-IRGs most relevant to HCC from common DE-IRGs and common IRGs most relevant to HCC.

The R package limma was utilized to determine the differential genes (DEGs) between the high-risk group and low-risk group among the three sets based on the filter condition (| log2FC |≥1, FDR <0.05). We nextran Gene Ontology (GO) and Kyoto Encyclopedia of Genes and Genomes (KEGG) to enrich biological functions and pathways related to common DE-IRGs and common DE-FRGs using the R package “org.Hs.eg.db”, respectively (Fan et al., 2021).

After setting the screening criterion of *p* < 0.05, we ran univariate COX regression analysis to screen prognostic differentially expressed immune-related genes (PR-DE-IRGs) and prognostic differentially expressed ferroptosis-related genes (PR-DE-FRGs) based on TCGA data after combining survival information, respectively. To rank the importance of the 29 PR-DE-FRGs as eigengenes, we ran the random forest algorithm based on the minimum points of cross-validation error using the R package “randomForest” (Fan et al., 2022b; Tian et al., 2022). Next, we screened 17 PR-DE-FRGs with an importance score >1 as the characteristic genes of HCC. After setting the correlation coefficient >0.3 and *p* < 0.001 as filtering conditions, we ran co-expression analysis based on 34 PR-DE-IRGs and 17 PR-DE-FRGs’ sequencing value for filtering PR-DE-IRFeCGs. We visualized the expression value of these 17 PR-DE-IRFeCGs using a heatmap, and visualized the co-expression network consisting of 17 PR-DE-IRFeCGs and the matching PR-DE-IRGs.

### Screening PR-DE-IRFeCGs for constructing prognostic predictive model

We integrated survival information and sequencing value from all TCGA samples, GSE10143 and ICGC.LIRI cohorts to obtain tissues that also covered these information. The clinical information of these tissues used for subsequent analysis was presented in Table 1. 370 TCGA HCC cases were randomly matched to the training and test sets in a 7 to 3 ratio. The optimal penalty parameter (*λ*) obtained based on the minimum 10-fold cross-validation of Lasso regression finally screened out three PR-DE-IRFeCGs (G6PD, RRM2, and PRKAA2) for constructing the prognostic predictive model.

**TABLE 1:** Clinical characteristics of each cohort.

		TCGA				GEO	ICGC
Covariates	Type	Whole cohort	Test set	Training set	*p*-value	GSE10143 cohort	LIRI cohort
Overall Survival	≤1,095	280 (75.68%)	85 (78.7%)	195 (74.43%)	0.4603	13 (16.25%)	199 (76.54%)
	>1,095	90 (24.32%)	23 (21.3%)	67 (25.57%)		67 (83.75%)	61 (23.46%)
Survival status	Alive	240 (64.86%)	63 (58.33%)	177 (67.56%)	0.1164	48 (60.00%)	214 (82.31%)
	Dead	130 (35.14%)	45 (41.67%)	85 (32.44%)		32 (40.00%)	46 (17.69%)
Age	≤60	177 (47.84%)	53 (49.07%)	124 (47.33%)	0.8484	-	55 (21.15%)
	>60	193 (52.16%)	55 (50.93%)	138 (52.67%)		-	205 (78.85%)
Gender	FEMALE	121 (32.7%)	38 (35.19%)	83 (31.68%)	0.595	-	68 (26.15%)
	MALE	249 (67.3%)	70 (64.81%)	179 (68.32%)		-	192 (73.85%)
Grade	G1	55 (14.86%)	21 (19.44%)	34 (12.98%)	0.2325	-	40 (15.38%)
	G2	177 (47.84%)	45 (41.67%)	132 (50.38%)		-	117 (45.00%)
	G3	121 (32.7%)	35 (32.41%)	86 (32.82%)		-	80 (30.77%)
	G4	12 (3.24%)	5 (4.63%)	7 (2.67%)		-	23 (8.85%)
	unknown	5 (1.35%)	2 (1.85%)	3 (1.15%)		-	0 (0.00%)
Stage	I	171 (46.22%)	48 (44.44%)	123 (46.95%)	0.8831	-	-
	II	85 (22.97%)	25 (23.15%)	60 (22.9%)		-	-
	III	85 (22.97%)	21 (19.44%)	64 (24.43%)		-	-
	IV	5 (1.35%)	1 (0.93%)	4 (1.53%)		-	-
	unknown	24 (6.49%)	13 (12.04%)	11 (4.2%)		-	-
T stage	T1	181 (48.92%)	50 (46.3%)	131 (50%)	0.9264	-	-
	T2	93 (25.14%)	29 (26.85%)	64 (24.43%)		-	-
	T3	80 (21.62%)	22 (20.37%)	58 (22.14%)		-	-
	T4	13 (3.51%)	4 (3.7%)	9 (3.44%)		-	-
	unknown	3 (0.81%)	3 (2.78%)	0 (0%)		-	-
M stage	M0	266 (71.89%)	79 (73.15%)	187 (71.37%)	1	-	-
	M1	4 (1.08%)	1 (0.93%)	3 (1.15%)		-	-
	unknown	100 (27.03%)	28 (25.93%)	72 (27.48%)		-	-
N stage	N0	252 (68.11%)	72 (66.67%)	180 (68.7%)	0.4836	-	-
	N1	4 (1.08%)	0 (0%)	4 (1.53%)		-	-
	unknown	114 (30.81%)	36 (33.33%)	78 (29.77%)		-	-

### Validation of the biological value of modeled genes in HCC

We again visualized the co-expression network consisting of three genes and the matching PR-DE-IRGs. We mapped the Kaplan Meier survival curve for showing the influence of these three genes’ expression on HCC patients’ survival probability. The ROC curves based on these three genes’ expression from HCC and normal tissues were used to assess their diagnostic value. To identify independent effects of these three genes on prognosis, we ran univariate and multivariate COX regression analyses.

We used GEPIA website to compare the differences of three PR-DE-IRFeCGs expression in model between HCC and normal tissues. The Human Protein Atlas database (HPA, proteinatlas.org) provided free immunohistochemical (IHC) staining images, which reflected the protein expression of these three genes in HCC tissues and normal liver tissues. We further verified the differential expression of these three genes between HCC and normal liver tissues by comparing these IHC staining images.

To further verify the differences in the transcription levels of these three genes between HCC and normal liver tissues, we further detected the relative mRNA expression levels of these genes by quantitative real-time PCR (QRT-PCR) experiment. The Ethics Committee of the People’s Hospital of Danyang (2022-09-041) approvaled this study and patients consented to specimen collection. 18 matched pairs of HCC and adjacent paracancerous tissues came from the subjects who underwent surgery. Table 2 showed the primer sequences of all genes.

**TABLE 2:** All primer sequences used in QRT-PCR experiment.

Gene	Forward primer	Reverse primer
β-Actin	TGG​CAC​CCA​GCA​CAA​TGA​A	CTA​AGT​CAT​AGT​CCG​CCT​AGA​AGC​A
G6PD	CCG​CAA​ACA​GAG​TGA​GCC​CTT​C	AGG​ACT​CGT​GAA​TGT​TCT​TGG​TGA​C
RRM2	CAC​GGA​GCC​GAA​AAC​TAA​AGC	TCT​GCC​TTC​TTA​TAC​ATC​TGC​CA
PRKAA2	ATC​CGA​AGT​CAG​AGC​AAA​CCG​TAT​G	AAG​CCA​GCA​GCA​GAA​CAG​GAA​C

Total RNA was isolated from tissues using the TransZol Up Plus RNA Kit (TRANS, Beijing, China). According to the manufacturer’s instructions, cDNA was synthesized by using HiScript^®^ III RT SuperMix for qPCR (+gDNA wiper) (Vazyme, Nanjing, China). QRT-PCR was performed using the Roche Light Cycler 96 Real-time Fluorescent Quantitative PCR System (Roche Applied Science, Mannheim, Germany) and Taq Pro universal SYBR qPCR Master Mix (Vazyme, Nanjing, China). After normalizing all measured values to relative expression levels of β-actin using the 2^−ΔΔCT^ method, we compared differences in the expression levels of G6PD, RRM2 and PRKAA2 between paired tissues using paired *t*-tests.

Human hepatoma cells (HuH-7 and 97H) and human normal hepatocytes (LO2) were purchased from Shanghai Cell Bank of Chinese Academy of Sciences. These three kinds of cells were cultured in DMEM (Gibco, Cat#C11995500BT). All media contain 10% Fetal Bovine Serum (Excell, Cat#FSP500) and 1% Penicillin-Streptomycin Liquid (Solarbio, Cat#P1400). All cells were cultured at 37°C in 5% CO_2_’s humidified incubator. The culture medium was changed every 24 h, and cells were passaged every 2–3 days. We again used QRT-PCR to detect the relative RNA expression of G6PD, RRM2 and PRKAA2 in these three cells. We further compared the relative RNA expression differences of these three genes between hepatoma cells and normal hepatocytes.

### Verification and comparison of prognostic predictive model’s performance

To assigning a risk score for each cases from the TCGA, GSE10143 and ICGC.LIRI cohorts, we apply the coefficients obtained by the lasso regression to the next formula: $Risk\;score=\sum \left(\mathrm{PR}-DE-IRFeCGs\;expression\;values\times corresponding\;coefficient\right)$ . HCC patients in each cohort were divided into high-risk group and low-risk group based on the median risk score of each cohort. After ranking the risk score of each tissue, we visualize the risk score and survival status of each tissue. We mapped Kaplan-Meier curve to show the differences in survival probabilities of patients between high and low risk groups in each cohort. Receiver Operating Characteristic (ROC) curve was used to evaluate the performance of prognostic predictive model in predicting the patients’ prognosis in each cohort. We ran COX regression again to test whether the risk score could independently affect the prognosis of patients with TCGA.

A small number of previous studies, including Zhang et al., Long et al., Wan et al., and Wang et al., have attempted to develop ferroptosis-related prognostic predictive models to predict the HCC patients’ prognosis. To further compare the superiority of our model against these models, we used ROC curve, Kaplan-Meier curve and C-index to test the performance of these models. After obtaining the corresponding performance test results for each model, we compared them.

### Deep validation of model performance

We used a heatmap to visualize each clinical feature for each sample and compared the differences in risk score between subgroups for different clinical features. Not only that, we also tested the ability of our model in distinguishing the prognosis of samples in each clinical feature subgroup.

### The guiding value of the model in clinical treatment

Since immunity and ferroptosis play an important role in cancer treatment, especially HCC, we further explored the predictive value of our prognostic predictive model in ICIs therapy and chemotherapy.

The key target genes’ expression of immune checkpoint blockade (ICB) have been shown to be associated with the clinical effects of ICIs (Hodi et al., 2010). For example, the expression of programmed death ligand 1 (PD-L1 or CD274) has gradually become an effective indicator of immunotherapy response (Hodi et al., 2010). Therefore, after analyzing the correlation between risk score and CD274 expression, we also compared its differences between different risk groups. The online website TIDE (tide.dfci.harvard.edu/) calculated the Tumor Immune Dysfunction and Exclusion (TIDE), Microsatellite Instability (MSI), Dysfunction, Exclusion scores of each TCGA HCC tissue for us. TIDE algorithm and MSI were also used by many bioinformatics studies to predict the potential response to ICB therapy (Jiang et al., 2018). The bar chart was used to show their correlation with risk scores/3 modeled gene expression. Their differences between different risk groups were also compared.

The R package “pRophetic” also predicted the half-maximal inhibitory concentration (IC50) of each TCGA HCC tissue for 8 chemotherapeutic drugs for the HCC’s treatment for us. The triangle plot were used to show their correlation with risk score/3 modeled gene expression. Their differences between different risk groups were also compared.

### Construction of a comprehensive quantitative nomogram for accurate prognostic prediction

Based on our superior model, we hope to further construct a quantitative tool that can integrate composite factors to accurately predict HCC patients’ prognosis. The comprehensive factors nomogram satisfies this need well. We integrated the clinical factors of the HCC tissues provided by TCGA, including risk groups, age, gender, grade and stage to draw the comprehensive factors nomogram. In this process, the R package “regplot” came into play. Next, the ROC curve and internal calibration curve were used to test the ability and accuracy of our nomogram in predicting prognosis.

### Statistical method

Student’s *t*-test was used to compare the differences of continuous variables that fitted a normal distribution between different groups, while a nonparametric test was used to compare the differences of continuous variables that did not fit a normal distribution between different groups. The chi-square test or Fisher’s exact test was used to compare the differences of categorical variables between different groups. R programming language (version 4.1.2) and Perl (version 5.8.3) provided free services for the statistical processing and plotting in this study. In all statistical treatments, unless otherwise specified, *p* < 0.05 was considered statistically significant.

## Results

### Identification of PR-DE-IRFeCGs

**FIGURE 2:**
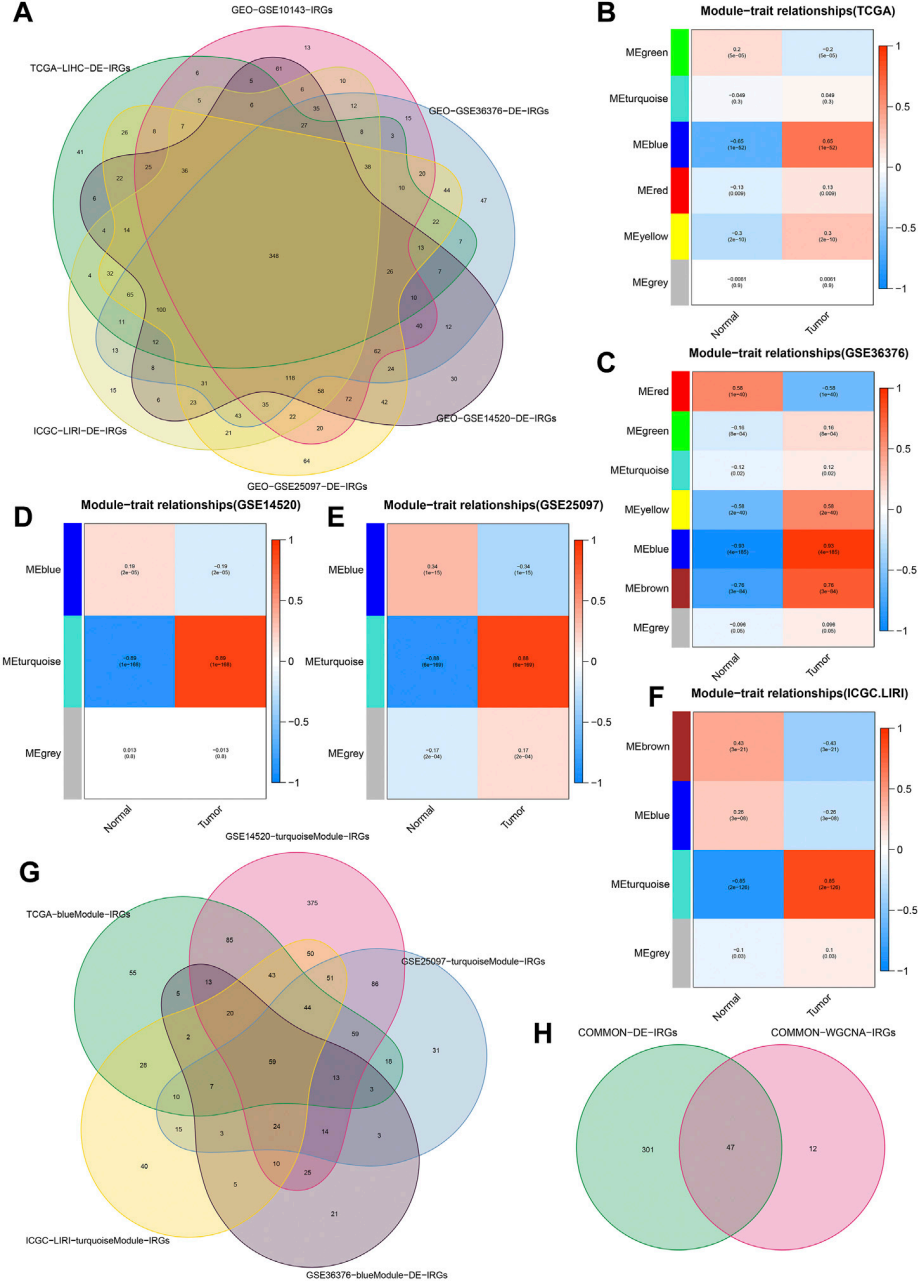
Identification of common DE-IRGs highly related to HCC. **(A)** The extraction process of common DE-IRGs from all the cohorts. **(B–F)** Heatmap showing the correlations between modules and HCC features in the TCGA, GSE36376, GSE14520, GSE25097, and ICGC cohorts, respectively. **(G)** The extraction process of common IRGs highly related to HCC. **(H)** The extraction process of common DE-IRGs highly related to HCC.

We obtained 192 DE-FRGs and 954 DE-IRGs from the TCGA cohort, 183 DE-FRGs and 1266 DE-IRGs from the GSE36376 cohort, 156 DE-FRGs and 1289DE-IRGs from the GSE14520 cohort, 178 DE-FRGs and 1491 DE-IRGs from the GSE25097 cohort, and 183 DE-FRGs and 1173 DE-IRGs from the ICGC.LIRI cohort respectively. Figure 2A showed the process of extracting 348 DE-IRGs via Venn diagram.

Based on the *β* = 4 of TCGA cohort, *β* = 7 of GSE36376 cohort, *β* = 3 of GSE14520 cohort, *β* = 6 of GSE25097 cohort and *β* = 8 of ICGC cohort, we identified the blue (Figure 2B), blue (Figure 2C), turquoise (Figure 2D), turquoise (Figure 2E) and turquoise (Figure 2F) modules with the strongest negative correlation with HCC, respectively. The Venn diagrams again extracted 59 common IRGs highly related to HCC (Figure 2G) and 47 common DE-IRGs highly related to HCC, respectively (Figure 2H).

**FIGURE 3:**
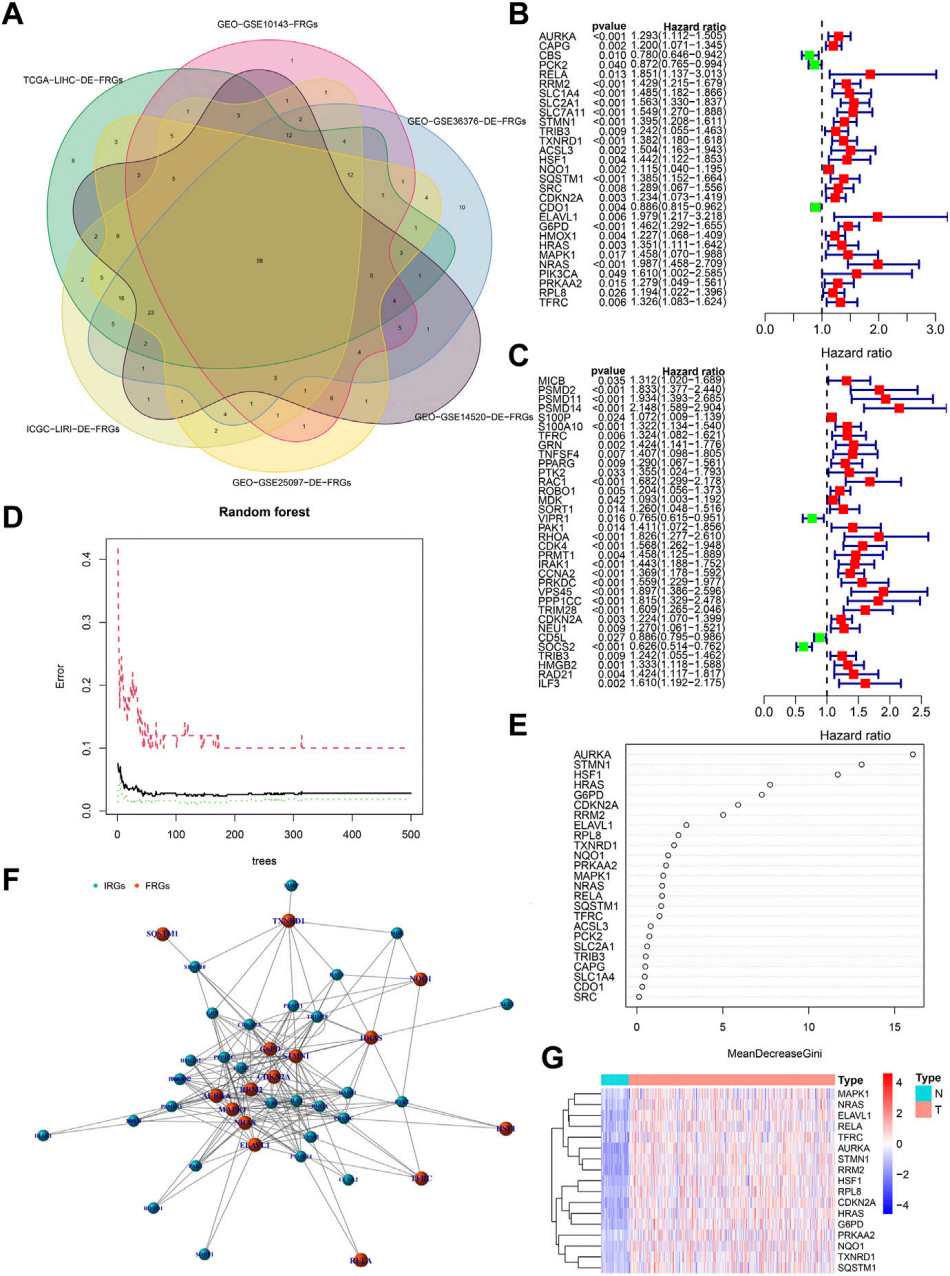
Identification of PR-DE-IRFeCGs. **(A)** The extraction process of common DE-FRGs from all the cohorts. **(B)** Forest plot showing the results of univariate COX regression analysis of 29 PR-DE-FRGs. **(C)** Forest plot showing the results of univariate COX regression analysis of 34 PR-DE-IRGs. **(D)** The influence of the number of decision trees on the error rate. The *x*-axis represents the number of decision trees and the *y*-axis is the error rate (Wu et al., 2022). **(E)** The importance score of the PR-DE-IRFeCGs based on the Random Forest algorithm (Wu et al., 2022). The PR-DE-IRFeCGs of the Gini coefficient method are based on random forest classifier. The *x*-axis represents the importance index, and the *y*-axis represents the genes (Wu et al., 2022). **(F)** The co-expression network between PR-DE-IRFeCGs and the corresponding PR-DE-IRGs. **(G)** Heatmap reflecting the expression levels of these 17 PR-DE-IRFeCGs.

Figure 3A showed the process of extracting 58 DE-FRGs via Venn diagram. The forest plots showed 29 PR-DE-FRGs and 34 PR-DE-IRGs identified by univariate COX regression (Figures 3B, C). The influence of the number of decision trees on the error rate was shown in Figure 3D. Figure 3E showed the relative importance scores of these PR-DE-FRGs ranked from top to bottom. Finally, we screened 17 PR-DE-FRGs with importance score greater than 1 as characteristic genes of HCC. Figure 3F visualized the co-expression network consisting of 17 PR-DE-IRFeCGs and the matching PR-DE-IRGs. The heatmap also showed the expression of these 17 PR-DE-IRFeCGs in HCC and normal paracancerous tissues (Figure 3G).

**FIGURE 4:**
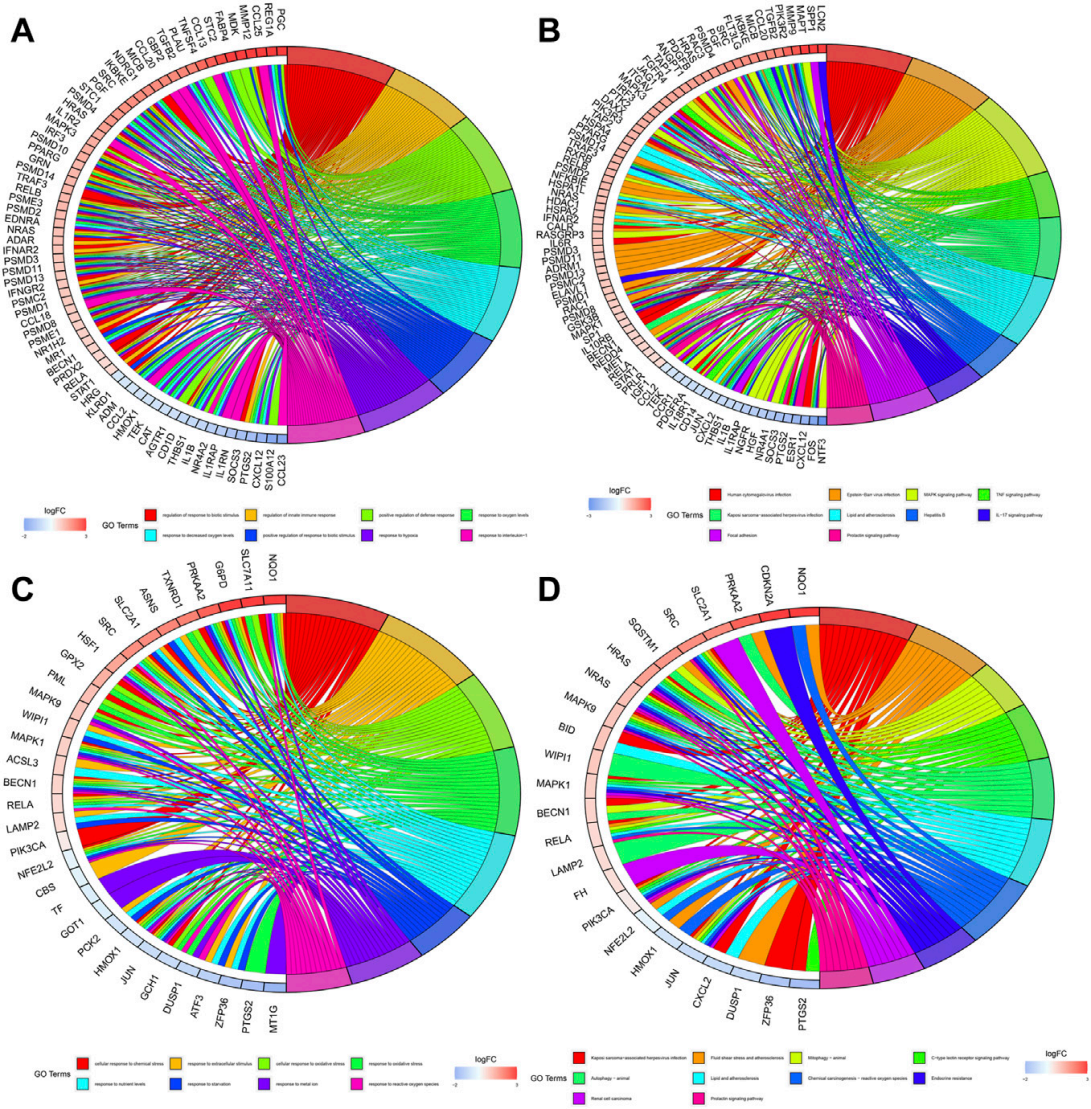
Enrichment of biological functions and pathways involved in common DE-IRGs and common DE-FRGs. **(A)** Biological functions involved in common DE-IRGs. **(B)** Biological pathways involved in common DE-IRGs. **(C)** Biological functions involved in common DE-FRGs. **(D)** Biological pathways involved in common DE-FRGs.

Figures 4A, B showed the biological functions and pathways that common DE-IRGs may be involved in, respectively. They are regulation of response to biotic stimulus, regulation of innate immune response, positive regulation of defense response, response to oxygen levels, response to decreased oxygen levels, positive regulation of response to biotic stimulus, response to hypoxia, response to interleukin-1, human cytomegalovirus infection, epstein-Barr virusinfection, MAPK signaling pathway, TNF signaling pathway, kaposi sarcoma-associated herpesvirus infection, lipid and atherosclerosis, hepatitis B, IL-17 signaling pathway, focal adhesion and prolactin signaling pathway. Similarly, Figures 4C, D showed the biological functions and pathways that common DE-FRGs may be involved in, respectively. They are cellular response to chemical stress, response to extracellular stimulus, cellular response to oxidative stress, response to oxidative stress, response to nutrient levels, response to starvation, response to metal ion, response to reactive oxygen species, kaposi sarcoma-associated herpesvirus infection, fluid shear stress and atherosclerosis, mitophagy-animal, c-type lectin receptor signaling pathway, autophagy-animal, lipid and atherosclerosis, chemical carcinogenesis-reactive oxygen species, endocrine resistance, renal cell carcinoma and prolactin signaling pathway.

### Screening PR-DE-IRFeCGs for constructing prognostic predictive model and validation of the biological value of modeled genes in HCC

**FIGURE 5:**
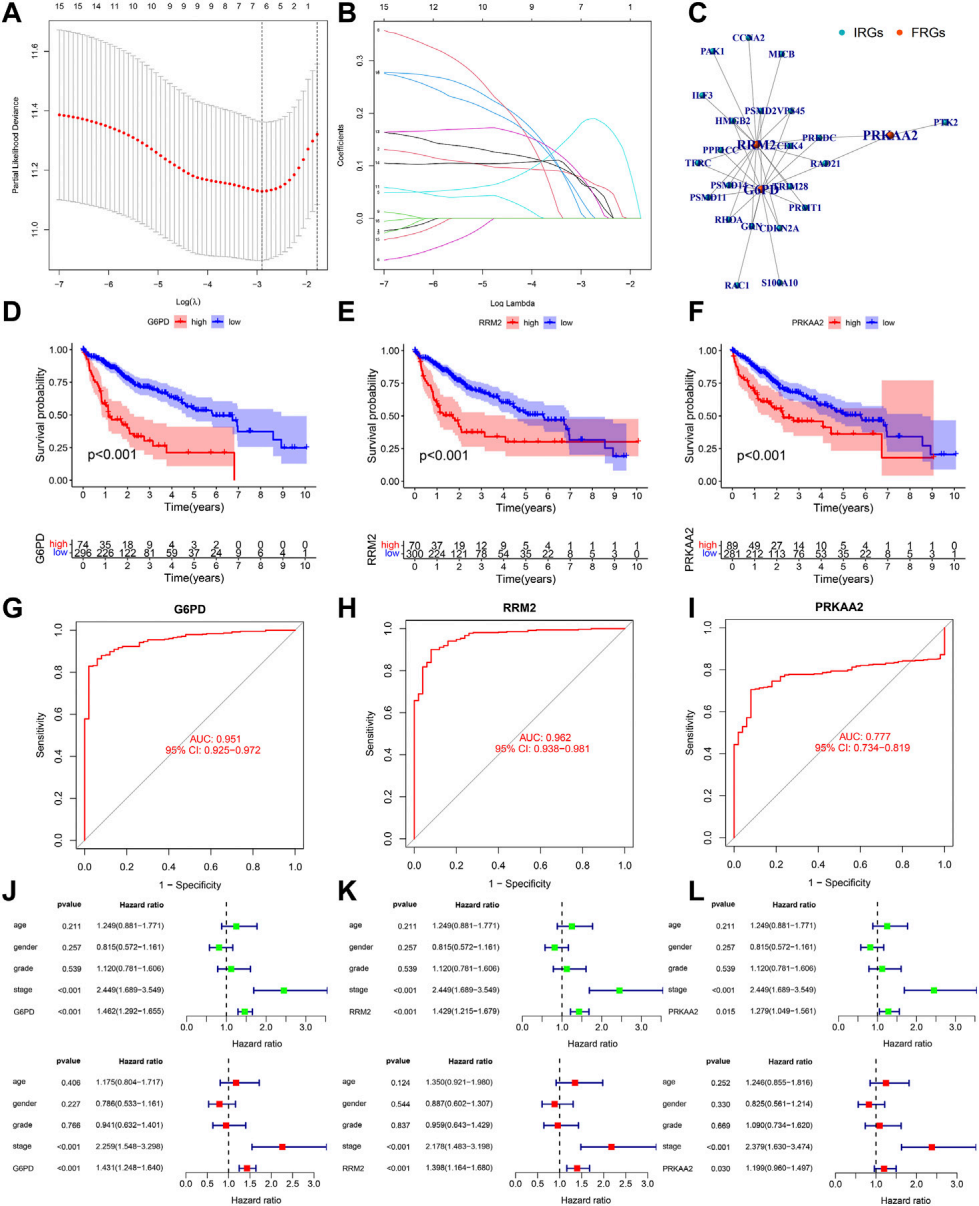
Validation of the biological value of modeled genes in HCC. **(A–B)** Lasso screening gene process. **(C)** The co-expression network between 3 modeled genes and the corresponding PR-DE-IRGs. **(D–F)** Kaplan Meier survival curves of G6PD, RRM2, and PRKAA2. **(G–I)** Diagnostic ROC curves of G6PD, RRM2 and PRKAA2. **(J–L)** Forest plots showing the results of univariate and multivariate COX regression of G6PD, RRM2, and PRKAA2.

Figures 5A, B showed the process of screening out three PR-DE-IRFeCGs and calculating the corresponding coefficients by Lasso regression. The co-expression network consisting of these three genes and the matching IRGs was shown in Figure 5C. Figures 5D–F showed that patients in the high expression group of the three modeled genes had lower survival probabilities. In the diagnostic ROC curves of all genes, the AUC values were greater than 0.75, suggesting that these genes have high diagnostic value in HCC (Figures 5G–I). Forest plots showed that these three modeled genes expression independently affected the HCC patients’ prognosis before and after adjusting for other clinical factors (Figures 5J–L).

**FIGURE 6:**
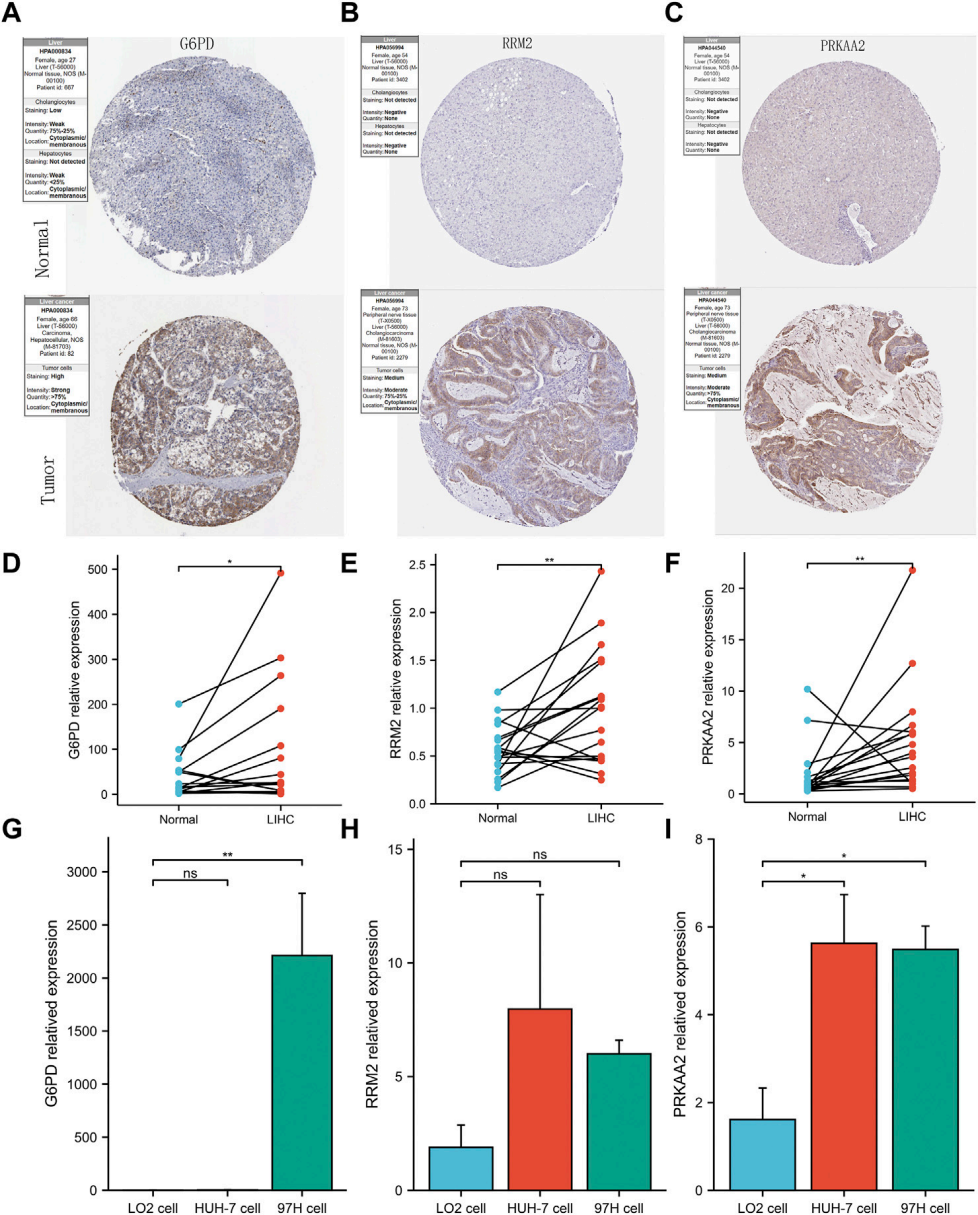
Validation of abnormal expression of 3 modeled genes in HCC. **(A–C)** IHC staining images from HPA reflecting the protein expression levels of G6PD, RRM2 and PRKAA2 in HCC/normal tissues. **(D–F)** Higher relative mRNA expression levels of G6PD, RRM2 and PRKAA2 detected by QRT-PCR in HCC tissues. **(G–I)** Higher relative mRNA expression levels of G6PD, RRM2 and PRKAA2 detected by QRT-PCR in Human hepatoma cells (HuH-7 and 97H).

To maintain the stability of the model, we tried to validate the differential expression of 3 genes in the model between HCC and normal liver tissues using data from an external database. GEPIA is a web tool server for cancer and normal gene expression profiling and interactive analyses (Tang et al., 2017). The boxplots from GEPIA showed that in addition to PRKAA2, the other two modeled genes were more highly expressed in HCC (Supplementary Figure S1A, B). The deeper staining of IHC suggests that the expression of the gene protein is higher. IHC staining images also showed that these three modelled genes had higher protein expression levels in HCC (Figures 6A–C). Not only that, the relative mRNA expression values of the three modeled genes detected by QRT-PCR were all higher in HCC tissues (Figures 6D–F). At the same time, we also observed that the relative RNA expression of these three genes in hepatocellular carcinoma cells was higher than that in normal hepatocytes (only G6PD and PRKAA2 showed significant statistical significance, Figures 6G–I).

### Verification and comparison of prognostic predictive model’s performance

**FIGURE 7:**
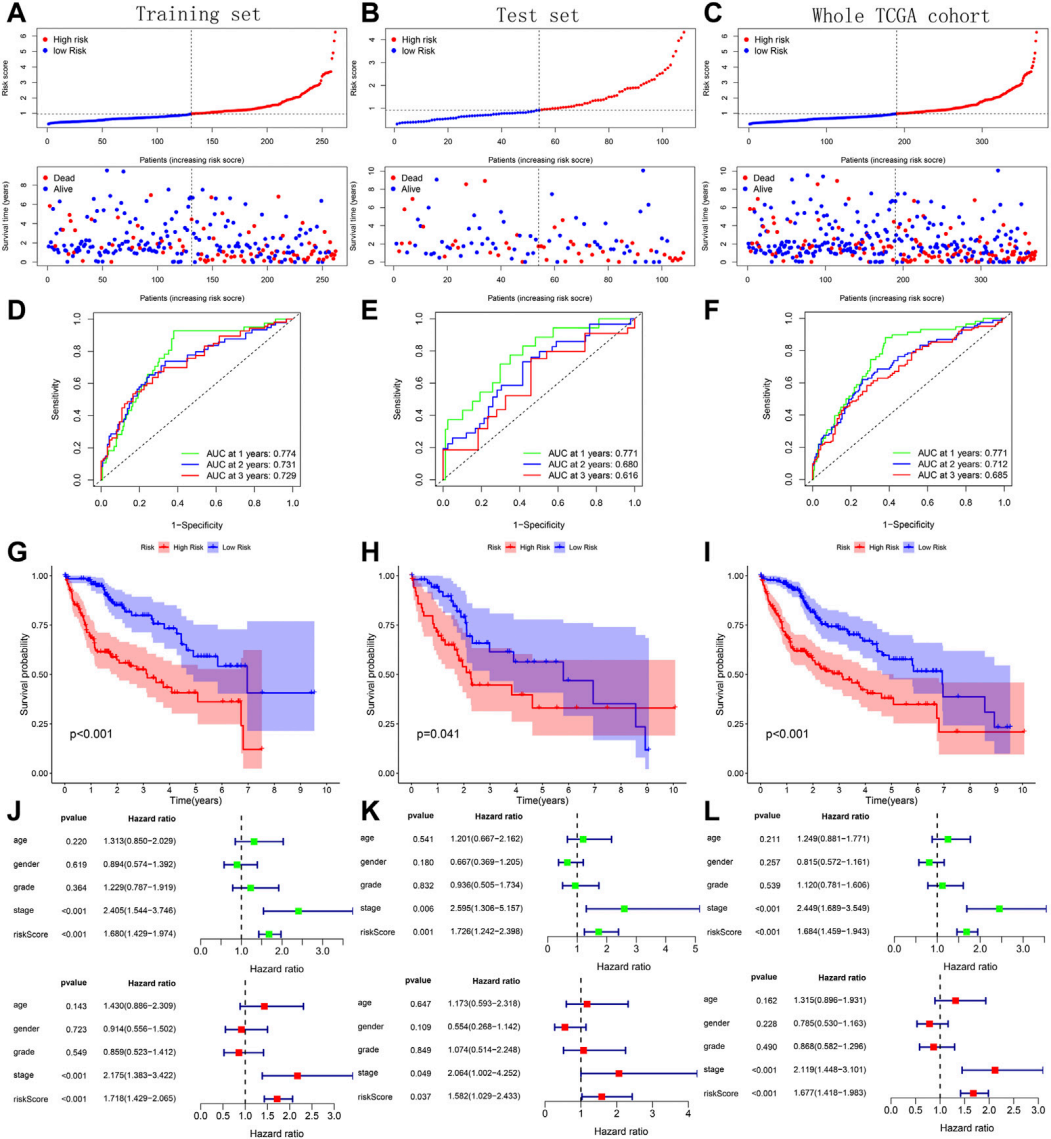
Verification of prognostic predictive model’s performance based on 3 TCGA sets. **(A–C)** Risk map and survival point map for the tissue of each TCGA set. **(D–F)** ROC curve based on the tissue of each TCGA set. **(G–I)** Kaplan Meier survival curve based on the tissue of each TCGA set. **(J–L)** Forest plot showing the results of univariate and multivariate COX regression of the tissue’s risk score in each TCGA set.

**FIGURE 8:**
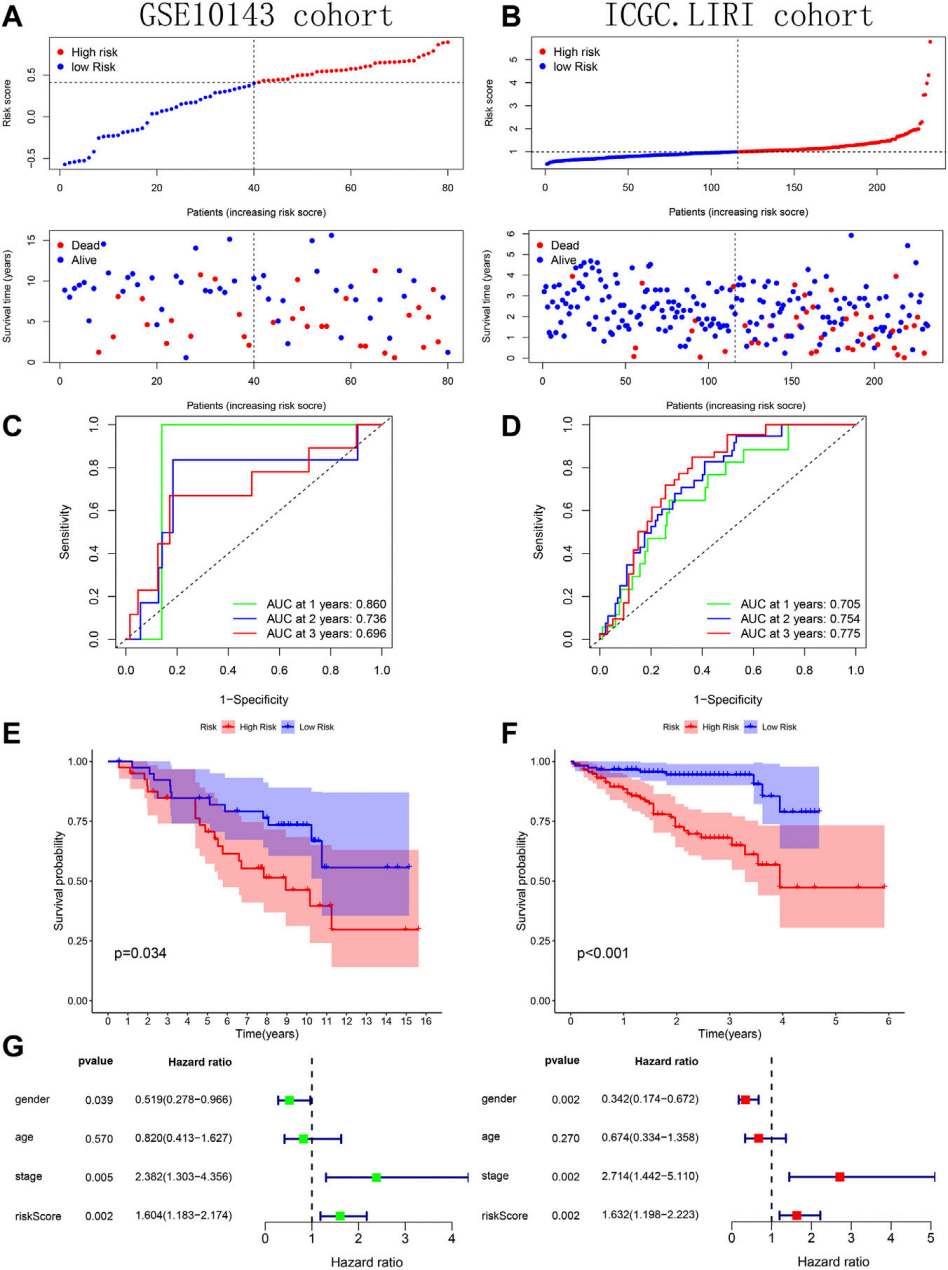
Verification of prognostic predictive model’s performance based on GSE10143 and ICGC.LIRI cohorts. **(A–B)** Risk map and survival point map. **(C–D)** ROC curve. **(E–F)** Kaplan Meier survival curve. **(G)** Forest plot showing the results of univariate and multivariate COX regression of the tissue’s risk score in GSE10143 cohort.

Across the 5 cohorts, there were significantly more deaths in the high-risk group (Figures 7A–C; Figures 8A, B). In the ROC curves of all cohorts, the AUC values were greater than 0.7 in most years, indicating that our model performed well in prognostic prediction (Figures 7D–F; Figures 8C, D). At the same time, we observed lower survival probabilities in the high-risk group samples (Figures 7G–I; Figures 8E, F). In the three sets of TCGA and GSE10143 cohorts, we found that risk scores could independently affect the HCC patients’ prognosis before and after adjusting for other clinical factors (Figures 7J–L; Figure 8G). These results fully showed that the samples in the high-risk group have a better outcome.

**FIGURE 9:**
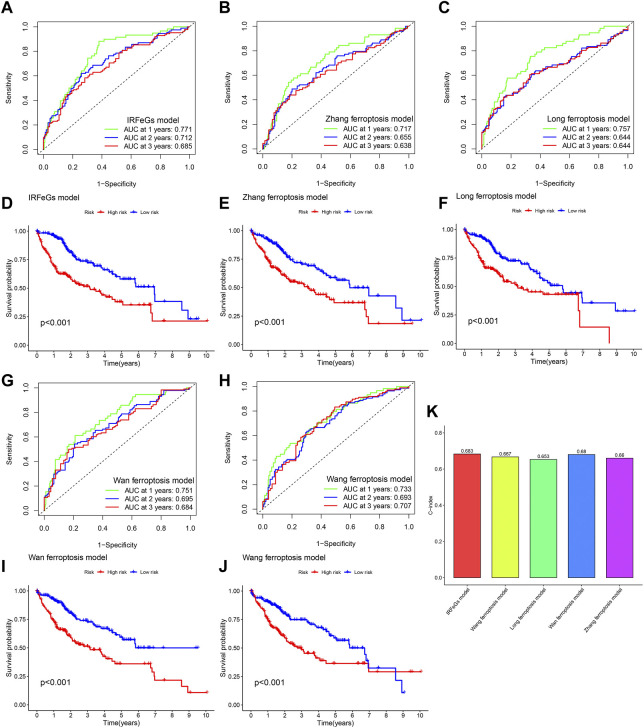
Comparison of prognostic predictive model’s performance based on TCGA cohort. **(A–C, G–H)** ROC curve based on the model of our IRFeCGs/Zhang et al./Long et al./Wan et al./Wang et al. **(D–F, I–J)** Kaplan Meier survival curve based on the model of our IRFeCGs/Zhang et al./Long et al./Wan et al./Wang et al. **(K)** C-index based on the model of our IRFeCGs/Zhang et al./Long et al./Wan et al./Wang et al.

Compared with the prognostic predictive models constructed by other studies, our model showed better performance in related tests. In almost every year, our prognostic predictive model had the highest AUC value (Figures 9A–C; Figures 9G, H). In addition, our model was slightly better than that of zhang et al., Long et al. and Wang et al. in distinguishing HCC tissues’ prognosis (Figures 9D–F; Figures 9I, J). Higher C-index is associated with better predictive performance of prognosis (Schröder et al., 2011). We also observed that the C-index of our model was higher than that of other models (Figure 9K). These test results strongly proved the superiority of our prognostic predictive model.

### Deep validation of model performance

**FIGURE 10:**
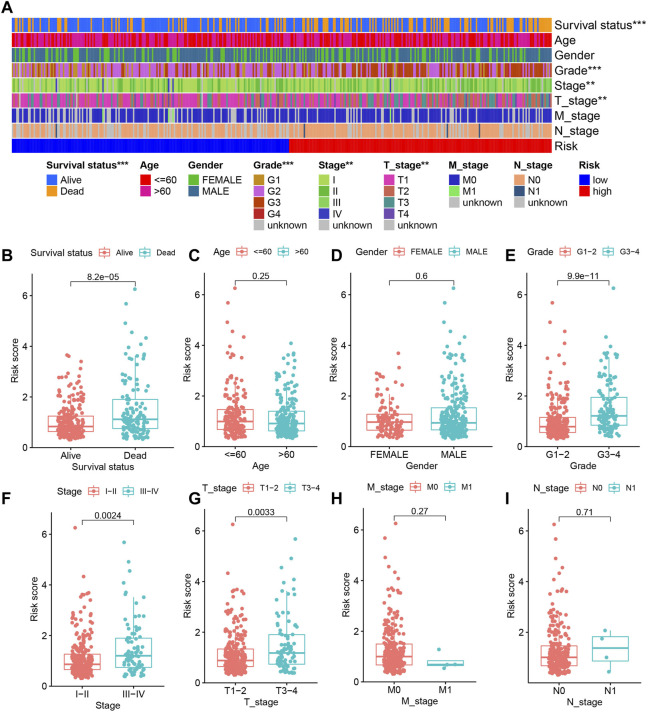
The relationship between risk score and clinical characteristics. **(A)** Heatmap showing the different clinical features of each TCGA tissue. **(B–I)** The differences in risk scores between different subgroups for each clinical characteristic.

**FIGURE 11:**
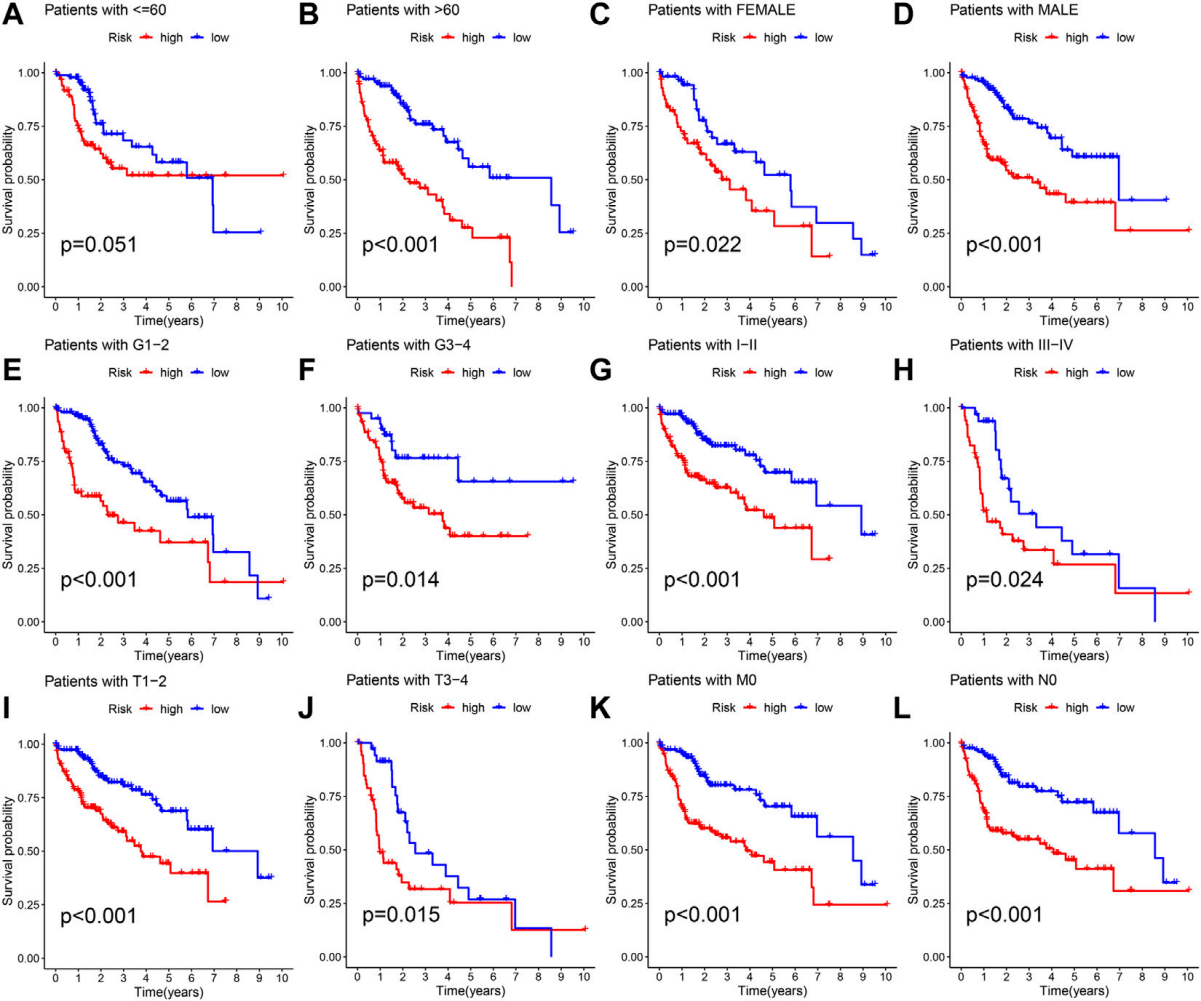
Deep validation of model’s performance. **(A–L)** The Kaplan Meier survival curves demonstrating the ability of the model to distinguish prognosis in different clinical subgroups.

The different clinical features of each TCGA tissue was visualized in Figure 10A. We also observed higher risk scores in the dead group, higher grade group, higher stage group and higher T satge group (Figures 10B–I). These results showed that the higher the malignant degree of the tumor in the high-risk sample. In addition, we observed that our prognostic predictive model maintained excellent ability to distinguish prognosis in other clinical subgroups except the age ≤60 years group (Figures 11A–L). There was no doubt that these results confirm that the model still had an excellent ability to distinguish prognosis in clinical subgroups.

### The guiding value of the model in clinical treatment

**FIGURE 12:**
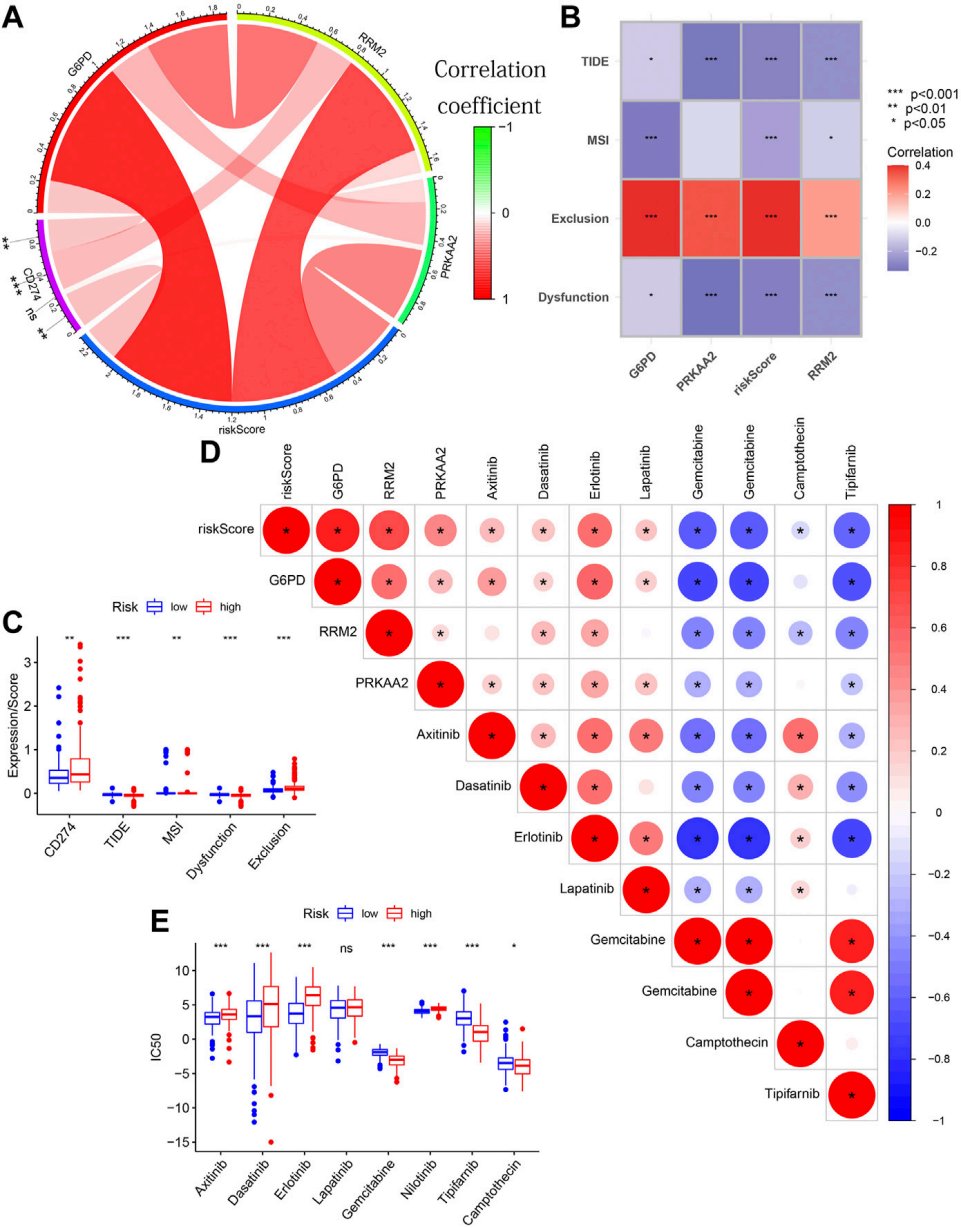
The guiding value of the model in clinical treatment. **(A)** The circle diagram showing the significant positive correlation between CD274 expression and G6PD expression/RRM2 expression/risk score. **(B)** The matrix diagram showing the negative correlation between TIDE score and the three modeling genes’ expression/risk score. **(C)** Boxplot showing the differences in CD274 expression/TIDE score/MSI score/Dysfunction score/Exclusion score between different risk groups. **(D)** The triangle plot showing a broad correlation between risk score/3 modeled genes’ expression and IC50 of eight chemotherapeutic drugs. **(E)** Boxplot showing the differences in the IC50 of eight chemotherapeutic drugs between different risk groups.

Tissues with higher CD274 expression and lower TIDE scores were considered to have favorable immune responses. The circle diagram showed a significant positive correlation between CD274 expression and G6PD expression/RRM2 expression/risk score (Figure 12A). The TIDE score showed a significant negative correlation with the three modeling genes’ expression/risk score (Figure 12B). From the box chart, we can see that the samples in the high-risk group have higher CD274 expression and lower TIDE score (Figure 12C). The results of further difference analysis also supported the results of the above correlation analysis. These results all suggested that tissues with higher risk score/G6PD’s expression/RRM2’s expression may benefit more in ICIs.

Figure 12D also showed a broad correlation between risk score/3 modeled genes’ expression and IC50 of eight chemotherapeutic drugs. Similarly, the results of further difference analysis also supported the results of these correlation analysis (Figure 12E). These results suggested that risk score/3 modeled genes’ expression can be used to predict the sensitivity of HCC patients to these 8 chemotherapeutic drugs. These results confirmed that the risk score was significantly correlated with the efficacy of immunotherapy and chemotherapy.

All in all, the above analysis results proved the potential guiding value of our prognostic predictive model in the HCC patients’ clinical treatment.

### Construction of a comprehensive quantitative nomogram for accurate prognostic prediction

**FIGURE 13:**
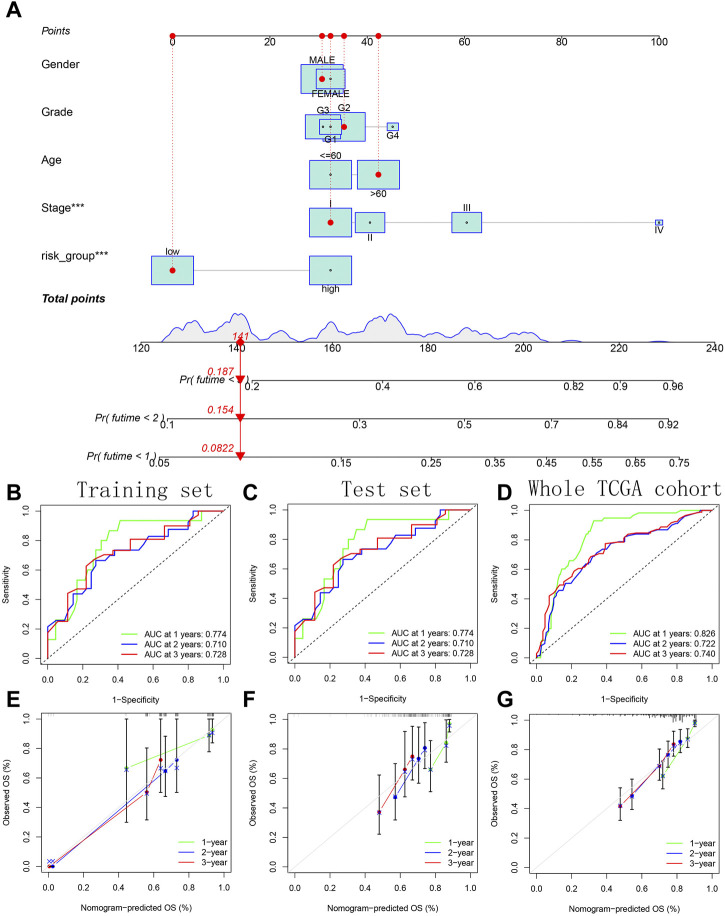
Construction and verification of a comprehensive quantitative nomogram. **(A)** The comprehensive quantitative nomogram quantifying various clinical indicators to predict the HCC patients’ survival probabilities. **(B–D)** The ROC curves confirming the good performance of nomogram in predicting survival probability. **(E–G)** The internal calibration curves confirming the prediction accuracy of nomogram.

A variety of potential prognostic clinical factors, including age, sex, clinical grade, clinical stage and risk group, were identified as constituent members of nomogram. As can be seen from comprehensive quantitative nomogram, we could quantify various clinical indicators to predict the HCC patients’ survival probabilities in 1-, 2-and 3-year (Figure 13A). The ROC curve confirmed the good performance of nomogram (Figures 13B–D). From the internal calibration curve, we observed that the predicted survival probability of comprehensive quantitative nomogram was basically consistent with the actual survival probability (Figures 13E–G).

## Discussion

HCC is both one of the most common cancers and a leading cause of cancer-related death (Pan et al., 2022). The main treatment methods for HCC include surgery, radiofrequency ablation, and biological therapy (Yao et al., 2021). Although some HCC patients are cured by partial hepatectomy, the overall survival outcome of HCC remains poor (Yao et al., 2021). The poor prognosis of HCC can be attributed to the fact that the diagnosis is usually made at an advanced stage of the cancer (Sun et al., 2020). Therefore, the development of optimal risk stratification scores and models is crucial to identify high-risk groups, which will benefit the surveillance and prevention of HCC (Shah et al., 2023). This study ran novel algorithms such as WGCNA and Random Forest to screen PR-DE-IRGs highly related to HCC and characteristic PR-DE-FRGs to run co-expression analysis for 17 PR-DE-IRFeCGs. Lasso regression further identified 3 PR-DE-IRFeCGs for us and constructed a prognostic predictive model. A series of analysis methods, including ROC curves, Kaplan-Meier survival curves and Cox regression, fully verified the diagnostic and prognostic value of modeling genes in HCC. GEPIA and IHC, QRT-PCR experiments further confirmed the upregulated expression of modeling genes in HCC. Our prediction model performed well in a variety of tests based on multiple cohorts. Not only that, it showed unique advantages compared with other related models. At the same time, it also showed outstanding guiding value in immunotherapy and chemotherapy response in patients with HCC. As a quantitative tool with repeatedly tested performance, the comprehensive quantitative nomogram we constructed could accurately predict HCC patients’ survival probability.

Although we have used multiple datasets, comprehensive online website, and experimental methods to fully verify the biological value of the three modeled genes in HCC, further support from a large number of literature reviews is still necessary. As a catalytic subunit of ribonucleotide reductase, RRM2 can significantly affect DNA replication and cell proliferation (Yang et al., 2021c). Numerous studies have observed that RRM2 is overexpressed in many cancers, including renal cell carcinoma (Xiong et al., 2021), colorectal cancer (Liu et al., 2013), lung cancer (Jin et al., 2020), bladder cancer (Morikawa et al., 2010), and head and neck cancer (Morikawa et al., 2010), and is regarded as a promoter for cancer progression and therapeutic target (Zhan et al., 2021). In addition, RRM2 have been reported in previous studies as an endogenous ferroptosis inhibitor, which maintains glutathione synthesis by regulating glutathione synthase, thereby exerting an anti-ferroptotic effect in HCC (Yang et al., 2020). G6PD, a key molecule involved in pentose phosphate pathway, has been reported to be involved in erastin-induced ferroptosis in non-small cell lung cancer cells. As an adverse prognostic factor, G6PD has also been observed to promote the progression of many types of cancer (Hu et al., 2013; Chen et al., 2018; Feng et al., 2020). PRKAA2, also known as AMP-activated protein kinase (AMPK), is an important energy-sensitive enzyme used to monitor the energy state of cells (Weijiao et al., 2021). It has been found that inhibition of AMPK can reduce the activity of GBM tumor cells (Chhipa et al., 2018). In addition, the high expression of PRKAA2 may indicate a poor prognosis in head and neck squamous cell carcinoma (Chhipa et al., 2018) and colorectal cancer (Zhang et al., 2020). Studies also have found that cancer cells with high basal AMPK activity are resistant to ferroptosis, and AMPK inactivation makes these cells sensitive to ferroptosis (Lee et al., 2020). These previous results are consistent with our results, which well confirm the biological role of the three modeled genes in cancer, especially those related to ferroptosis. The significant biological value of these genes in cancer also fully supports the stability of gene sources in the construction of our prognostic predictive model.

We have observed that several previous studies had focused on the identification of ferroptosis-related genes signature in HCC, including signature of five ferroptosis-related genes constructed by Zhang et al. (2022), the signature of four ferroptosis-related genes constructed by Long et al. (2022), the signature of five ferroptosis-related genes constructed by Wan et al. (2022), and the signature of seven ferroptosis-related genes constructed by Wang et al. (2022). It is worth mentioning that these signature have their own advantages. Unfortunately, they all focused solely on ferroptosis and ignored the immunity that often coexists with ferroptosis. Our study also focused on the identification of PR-DE-IRGs, PR-DE-FRGs, and PR-DE-IRFeCGs, which is a novelty from these studies and is more in line with the synergy of ferroptosis and immunity in cancer progression. Obviously, we extensively used 5 datasets from 3 databases to identify common DE-IRGs and DE-FRGs, which well guaranteed the accuracy of the analysis results. In the identification of DE-IRGs highly related to HCC, we also used a novel algorithm-WGCNA based on multiple datasets respectively. And we further used the machine learning algorithm-random forest to screen characteristic PR-DE-FRGs in HCC. These are not covered in other studies. In the end, we used the minimum number of genes among several signatures to conveniently and efficiently construct this novel signature. To ensure the stability of the gene source of the model, we also fully verified the significant biological value of the three modeled genes in HCC through a variety of methods, including several experimental methods. We also observed that the most performance test cohort and depth test methods were used in our study, which more fully confirmed the superior performance of our signature. Surprisingly, our model also performed the best in the corresponding tests, which was not only reflected in the ROC curve and C-index, but also in the part of Kaplan-Meier curve. In the field of clinical application, our model and 3 modeled genes showed significant guiding value in almost all immunotherapy and chemotherapy responses, which was also superior to other models.

Although we have identified and verified a novel immune-related ferroptosis signature with excellent predictive performance and clinical guidance value through complex bioinformatics methods, this study still has many limitations. Due to the great difficulty of collecting relevant data in clinical practice, the performance of the model still lacks verification of data from the latest clinical tissues. At the same time, the limited data types limited the in-depth validation of the model.

## Data Availability

The original contributions presented in the study are included in the article/Supplementary Material, further inquiries can be directed to the corresponding author.

## Supplementary material

SUPPLEMENTARY FIGURE S1 Validation of abnormal expression of 3 modeled genes in HCC based on GEPIA database. **(A–C)** GEPIA boxplots showing the differences of G6PD, RRM2 and PRKAA2 expression between HCC and normal tissues.
