# Supplementary figures and images for: Identification and verification of novel immune-related ferroptosis signature with excellent prognostic predictive and clinical guidance value in hepatocellular carcinoma
Source: Front Genet. 2023 Aug 21;14:1112744. doi: 10.3389/fgene.2023.1112744 (PMC10475594; doi:10.3389/fgene.2023.1112744)

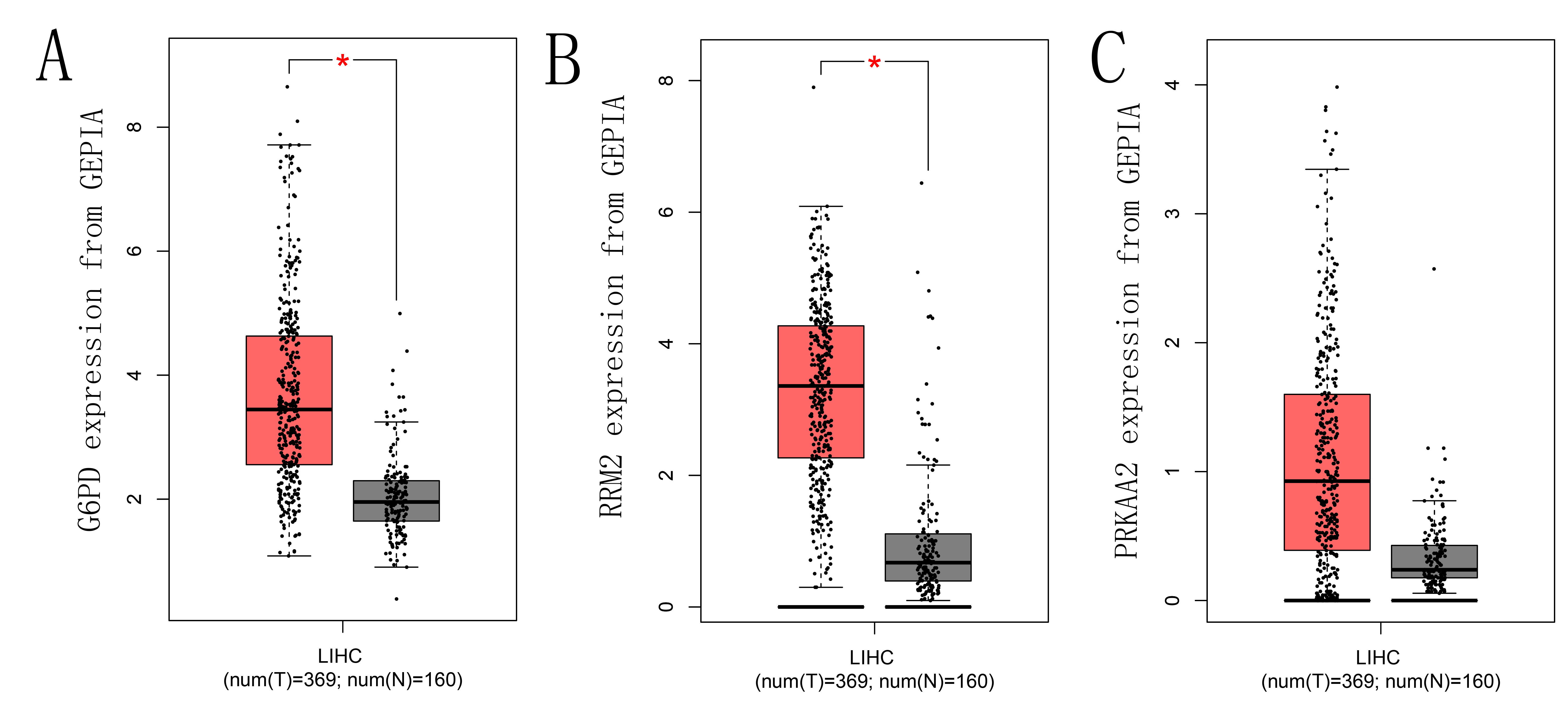

Supplement: Supplementary file 1 [file Image1.JPEG]
